# Management of Venous and Arterial Thrombosis

**DOI:** 10.3390/jcm13102744

**Published:** 2024-05-07

**Authors:** Lucia Stančiaková, Maha Othman, Peter Kubisz

**Affiliations:** 1 National Centre of Haemostasis and Thrombosis, Department of Haematology and Transfusion Medicine, Jessenius Faculty of Medicine in Martin, Comenius University in Bratislava, Martin University Hospital, 036 59 Martin, Slovakia; peter.kubisz@uniba.sk; 2 Department of Biomedical and Molecular Sciences, School of Medicine, Queen’s University, Kingston, ON K7L 3N6, Canada; othman@queensu.ca

A thrombus is a hemostatic plug localized in a blood vessel. This blockage might lead to partial or complete obstruction of blood flow in arteries, veins, or microcirculation [1].

Acute myocardial infarction (MI) is mainly a consequence of coronary atherosclerosis, and it is caused by thrombotic occlusion of an arterial lumen [2]. The pathology of myocardial infarction (MI) is divided into ST-elevation MI (STE-MI) and non-ST-elevation MI (NSTE-MI). MI is a major cause of human death, and more than 3 million people develop STE-MI every year. Although the worldwide rate of MI-related mortality has decreased, the incidence of heart failure is still high [3]. Moreover, one of the complications is a thromboembolism precipitated by left-ventricular thrombosis based on chronic dysfunction in this area [4]. One of the ways of reducing the prevalence of MI-related disorders is to identify high on-treatment platelet reactivity, which is still challenging and often inaccurately identified [5]. 

Stroke is also a prevalent vascular disorder that leads to significant morbidity and mortality of affected individuals. Biomarkers including C-reactive protein, interleukins 6 and 10, low-density lipoprotein cholesterol, total cholesterol, and homocysteine can serve as predictors of cognitive decline after this ischemic complication [6]. Congenital heart abnormalities might contribute to microthrombi formation and valvular incompetence, potentially eventuating in embolization as well [7]. 

Deep venous thrombosis (DVT) is a blood clot developed in non-superficial veins. Thus, venous thromboembolism (VTE) refers to an in situ thrombus and a dislodged thrombus—an embolus occurring predominantly in the lungs and serving as a life-threatening pulmonary embolism (PE) [1]. VTE is the third-most-frequent underlying cause of death. Its incidence is estimated at 1.43 per 1000 people a year; for DVT, it is 0.93 per 1000 people a year, and for PE, it is 0.50 per 1000 people a year. Due to improvements in the diagnostics for and treatment of VTE in the last few decades, there has also been a decrease in VTE-associated deaths, plummeting from 12.8 to 6.5 deaths per 100,000 people [8]. However, VTE can lead to further clinical conditions with significant morbidity and mortality, including the extension of thrombi, recurrence, chronic thromboembolic pulmonary hypertension, and post-thrombotic syndrome [9]. 

One of the clinical manifestations of thrombosis at the level of microcirculation is the pregnancy loss caused by impaired placental blood flow. Such complication might be caused by various prothrombotic conditions including enhanced platelet activation playing the role in the pathogenesis of the sticky platelet syndrome [10].

A strategy consisting of D-dimer testing with a borderline increasing with age has been developed for diagnosing VTE [11]. Assessment of D-dimer levels has a high negative predictive value, with a negative test indicating an absence of thrombosis in an organism [12]. Unfortunately, an elevated concentration of D-dimers is nonspecific, especially in cancer patients. The high prevalence of VTE in this population decreases its negative predictive value and undermines pretest probability assessed via the Geneva or Wells scores otherwise used to guide evaluation for a PE [13].

The Pulmonary Embolism Severity Index (PESI) and its simplified version are designed to distill clinical information, including vital functions and comorbidities, into a risk score. The PESI score indicates increased all-cause mortality at 30 days after a diagnosis of PE. Its simplified form (sPESI) has limited specificity in predicting mortality among high-risk patients. Risk indicators for PE include serologic markers of right-ventricular dysfunction and myocardial injury, echocardiography, computed tomography pulmonary angiography (CTPA), and the evaluation of hemodynamic status via right-heart catheterization [14].

Anticoagulant drugs are used in the acute (the first week), long-term (7 days up to 3 months), and extended (3 months and longer) treatment of VTE. Anticoagulation can be managed with low-molecular-weight heparin (LMWH), fondaparinux, unfractionated heparin (UFH), direct oral anticoagulants (DOACs), and/or vitamin K antagonists (VKAs). Deciding which type of anticoagulant to use depends on the indication, the underlying condition, the preference of the patient, and bleeding risk [15], as assessed using various strategies of VTE prophylaxis. For most of these drugs, under specific occasions, we can test their effectiveness that might be associated with a lower rate of thromboembolic episodes [16]. Moreover, there are novel treatment options for both arterial and venous thromboembolic episodes, such as proprotein convertase subtilisin/kexin type 9 (PCSK9) inhibitors that decrease the levels of lipoprotein (a) [17,18].

Systemic thrombolytic therapy reduces pulmonary arterial pressure and length of hospitalization [14]. In severe cases of iliofemoral DVT, catheter-directed thrombolytic systems and mechanical thrombectomy show promising results not only in terms of decreasing the risk of PE development and death but also in terms of ameliorating its later consequences [19]. Such endovascular treatment might be a rescue option for patients with a deteriorating clinical state or those for whom standard forms of therapy failed or was associated with contraindications [20].

Along with activated epithelium, neutrophils produce pro-inflammatory cytokines and chemokines to promote local inflammation. Under the conditions of sustained activation, aside from the potential effect of circulating free hemoglobin, heme, and iron, they contribute to the release of neutrophile extracellular traps (NETs) [21,22]. Such NETs are associated with hypofibrinolysis and correlate with elevated lactate levels that indicate increased mortality in patients with acute PE [23]. Increased serum lactate levels are also a promising biomarker of the prognosis of patients with acute mesenteric ischemia [24].

One of the infections associated with inflammation and an increased risk of thromboembolic episodes is coronavirus disease 2019 (COVID-19) caused by SARS-CoV-2 infection [25]. However, the optimal dose of anticoagulants and the need for additional antiplatelet treatment for critically ill patients with COVID-19 require further investigation [26].

We, the Editors, are honored to have received so many high-quality articles addressing these and other topics written by recognized experts in the field of thrombosis and hemostasis. The excellence of these articles was verified by positive reactions and their increased number of citations to date. Predominantly, we sincerely hope that the information included in the articles of this Special Issue will help to improve the management of patients with thromboembolisms, thus increasing their quality of life. We hope that the readers will enjoy it as well.
